# Knowledge sharing in the health scenario

**DOI:** 10.1186/1479-5876-12-S2-S8

**Published:** 2014-11-28

**Authors:** Magí Lluch-Ariet, Albert Brugués de la Torre, Francesc Vallverdú, Josep Pegueroles-Vallés

**Affiliations:** 1 Barcelona Digital Technology Centre, Roc Boronat, 117, 08018, Barcelona, Catalonia, Spain; 2 Departament d'Enginyeria Telematica (ENTEL), Universitat Politècnica de Catalunya Jordi Girona, 1-3, C3, 08034 Barcelona, Catalonia, Spain; 3 University of Applied Sciences Western Switzerland Sierre, Switzerland

**Keywords:** Network Protocols, Distributed Systems, Multiagent systems, Data sharing

## Abstract

The understanding of certain data often requires the collection of similar data from different places to be analysed and interpreted. Interoperability standards and ontologies, are facilitating data interchange around the world. However, beyond the existing networks and advances for data transfer, data sharing protocols to support multilateral agreements are useful to exploit the knowledge of distributed Data Warehouses. The access to a certain data set in a federated Data Warehouse may be constrained by the requirement to deliver another specific data set. When bilateral agreements between two nodes of a network are not enough to solve the constraints for accessing to a certain data set, multilateral agreements for data exchange are needed.

We present the implementation of a Multi-Agent System for multilateral exchange agreements of clinical data, and evaluate how those multilateral agreements increase the percentage of data collected by a single node from the total amount of data available in the network. Different strategies to reduce the number of messages needed to achieve an agreement are also considered. The results show that with this collaborative sharing scenario the percentage of data collected dramaticaly improve from bilateral agreements to multilateral ones, up to reach almost all data available in the network.

## Introduction

Clinicians and biomedical researchers often need to compare the information collected from the exams performed on their patients with information from similar patients in other places. This is needed for accurate diagnosis, prognosis and theragnosis and an effective management of diseases. Providing mechanisms to facilitate the access to remote worldwide distributed data sets becomes relevant to foster collaboration and knowledge sharing.

When all the ethical and legal regulations to protect the clinical data are satisfied a negotiation process for data exchange can start. A clinician may add some constraint and give access to the data only if a certain set of conditions are satisfied. One typical constraint may be that another dataset is provided in return. Bilateral agreements between two clinical centres will not always solve all those constraints and involving a set of centres in multilateral agreements for data exchange would increase the amount of data potentially accessible in the network.

In this article, we provide the details of the implementation and the evaluation of a system (*MOSAIC*) for the finding of paths involving a set of nodes that all together can participate in a multilateral agreement for data exchange and knowledge sharing.

In this first section we have introduced the system developed, and the justification why it is needed. In the following section (The MOSAIC System), the components of the system and the negotiation process for the multilateral data exchange are explained. In the MOSAIC implementation section we explain the details of the implementation and the path selection for the network exploration. In the Validation and performance evaluation section we present the results and findings after analysing the results of the protocol execution in the proposed scenario. Finally, we summarise the main achievements and outline the future work to be done.

### State of the art

Public regulations push to open up and share data, for scientific publications [1] (Open Data), for research data [2] and also for clinical data [3]. Aiming to extract the maximum amount of knowledge from the data, a global alliance [4] for sharing genomic and clinical data was created on june 2013.

Well established interoperability standards for clinical information (DICOM [5], HL7 [6] or ISO/EN 13606 [7]), medical reference terminologies (SNOMED [8,9]), clinical ontologies, Electronic Health Records [10], Multi-Agent systems [11-13] and systems like PhysioNet [14] or caCORE [15], are facilitating the data transfer and interchange between clinical centres around the world. Ontologies to facilitate reasoning and support the trading of services over Internet are being designed (e.g. Linked USDL [16]). Nevertheless, it is still difficult to find and gain access to the best dataset for a certain purpose and none of these systems and technologies facilitate the finding of multilateral agreements for accessing to the desired data.

An example of a federated Data Warehouse and its associated Decision Support System is the HealthAgents project [17,18], that aims to build a system to manage a network of clinical centres for the brain tumour diagnosis. This project was focused on collecting data for building classifiers that would be used during the diagnose process, but did not address the exchange of data between the nodes.

The problem of solving multilateral agreements includes to the problem of finding the shortest path in a complex network. This could be rapidly solved using the Dijkstra algorithm [19]. However, this is possible only if the links between the nodes are known and the topology of the whole network is known. In many scenarios the information of the network topology is neither available nor complete. As an example of this, a clinician may accept to publish the reference of which datasets are available from his local repository, but the specific permission to allow access to them may not be provided before an explicit data access request from a specific centre is received. Thus, a centralised approach to solve this problem is not feasible and a distributed and dynamic mechanism for the exploration of the paths associated to possible multilateral agreements is needed.

## The MOSAIC System

### The components of the system

**Figure 1:**
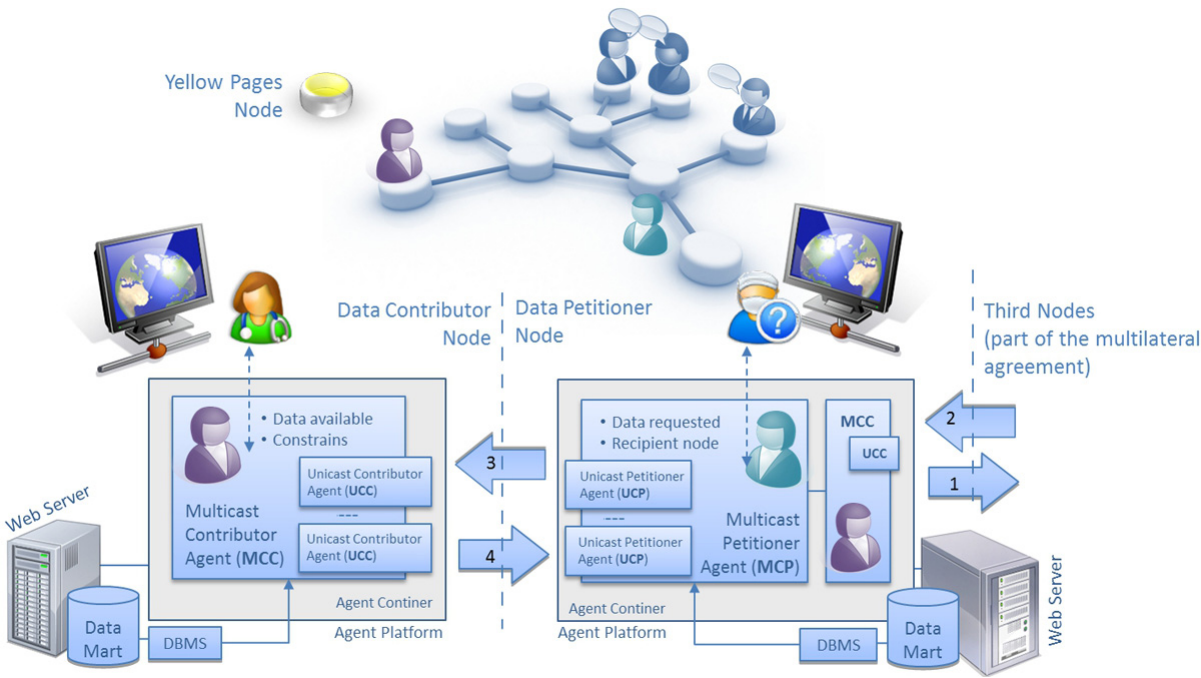
**The Architecture of the system**. The architecture of the *MOSAIC *System, showing the data flow among the agents. The Data Petitioner Node solves the constraint: 1) data delivery to third nodes, 2) data collection from third nodes needed to fulfill a constraint and 3) delivery of data requested. The Data Contributor node concludes the transaction: 4) delivery of the data requested.

The agent oriented abstraction fits well in the knowledge sharing scenario due to its distributed and dynamic nature. MOSAIC (see Figure 1) is a Multy-Agent System that facilitates the multilateral data exchange in a network by providing mechanisms for the intelligent search of paths to reach the datasets requested, involving a set of nodes in multilateral agreements. The MOSAIC System is composed by a set of interconnected nodes each one with its associated Data Mart and the Agent Platform to host the following Agents:

• **Multicast Contributor Agent (MCC)**. An Agent activated by the user to offer a certain dataset to the network, with or without constraints.

• **Unicast Contributor Agent (UCC)**. An Agent activated by the MCC to negotiate a specific data access request sent by an MCP.

• **Multicast Petitioner Agent (MCP)**. An Agent activated by the user or by a Unicast Petitioner Agent. The user launches it in order to explore the network looking for a certain data set. The UCP launches it in order to solve a constraint from a UCC when the dataset requested is not available at the node of the UCP.

• **Unicast Petitioner Agent (UCP)**. An Agent activated by the MCP in order to negotiate a specific data access request with a UCC.

• **Yellow Pages Agent (YP)**. An Agent that provides the directory service and hosts the list of references of MCCs active in the network.

**Figure 2:**
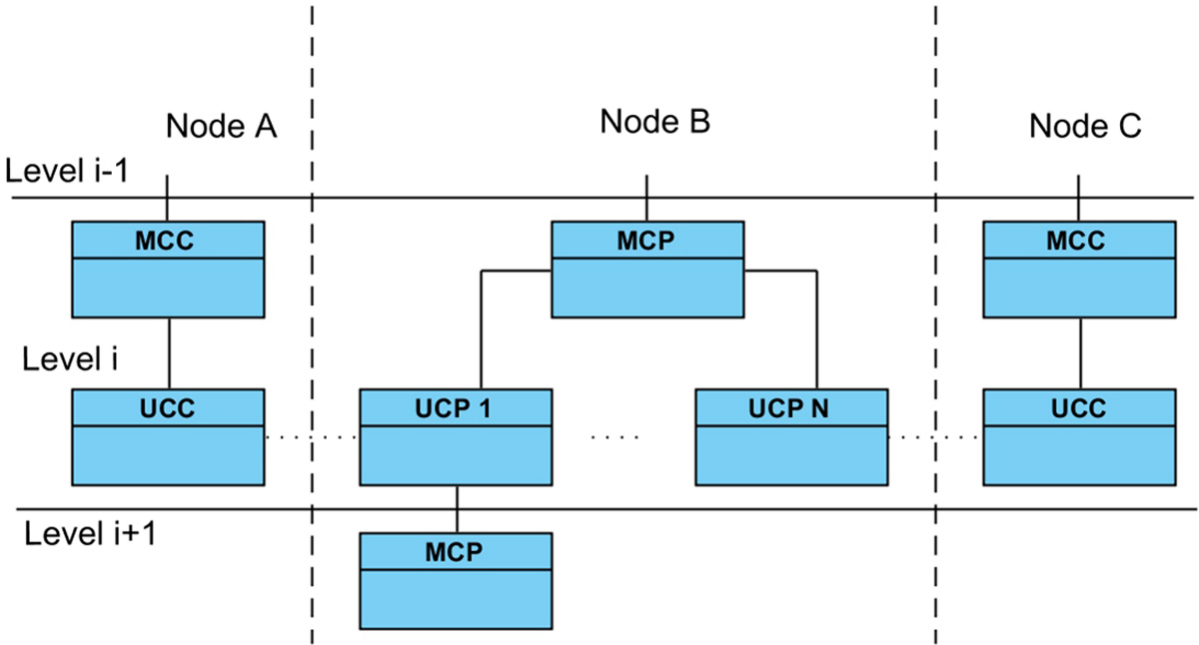
**The Agents of the system**. The MOSAIC Agents, their dependency and interactions during the execution of the protocol.

Figure 2 shows the dependences and relationships between the *MOSAIC *agents. Every link in a multilateral agreement is composed of two pairs of MCP-UCP and MCC-UCC.

### The negotiation process

From the launch of a data set request by the user, followed by the intermediate steps to solve the possible constraints, to the final delivery of the data, the *MOSAIC *protocol follows a process with the following five stages:

• **Stage 1: Network exploration**. After the activation of a MCP by the user the process to find paths that connect the requesting node with the ones hosting the desired data starts. This exploration ends with the identification of a set of nodes connected with the initiator (directly or with intermediate connections with other nodes).

• **Stage 2: Agreement proposal notification**. Every agent participating in a successful path will notify its creator about the possible agreement. At the end of this stage the initial MCP will receive a list of all existing possible agreements for the data exchange (corresponding to a list of paths that go from the leaf to the initiating MCP.).

• **Stage 3: Agreement selection and notification**. The MCP will select a path or a set of paths and notify this decision to all the agents involved, considering - among other criteria - to avoid overlapping agreements that solve the access to the same dataset of the same MCC through different paths, or to datasets already collected.

• **Stage 4: Data transfer**. After receiving the notification that a possible agreement is selected, the data exchange between all the nodes starts. This may end with a complete and successful data exchange or with some failure by some nodes. All the UCP waiting to receive data will send a message of acknowledgement (ACK) to their MCP after receiving the data or a message of non-acknowledgement (NACK) in case of a failure of data reception. The ACK (or NACK) is transmitted link to link until arriving to the main MCP at the top of the path.

• **Stage 5: Transaction completion**. After receiving all the ACK from all the nodes involved in the agreement, the initiating MCP will send a COMMIT to all the Agents. In case some ACK is not received or a NACK is transmitted by some Agent, the MCP will send a ROLLBACK message to all the nodes. Only after the reception of a COMMIT the nodes will have the authorisation to use the data received. In case the transaction is aborted with a ROLLBACK, none of the nodes of an agreement that received data are authorised to use it.

During the negotiation process the agents generate a number of messages (*N *) and those that correspond to the communications among different nodes involved in a multilateral agreement (*i*), follow the calculation of Eq. (1).

(1) $$N_{i}=m_{i}\left(4u_{i}+2\right)$$

Where *m_i _*and *u_i _*are the number of MCP and UCP respectively, involved in the agreement *i*. The four messages per UCP correspond to the following:

• **MCP ***→ ***MCC**: Dataset request

• **UCC ***→ ***UCP**: Notification of the constraint

• **UCP ***→ ***UCC**: Constraint delivery

• **UCC ***→ ***UCP**: Acceptance of the agreement

The two messages per MCP correspond to:

• **MCP ***→ ***YP**: Request from the MCP to the YP asking for the MCC offering the desired dataset

• **YP ***→ ***MCP**: Response from the YP to the MCP with the list of references of MCC available

This measure is useful for the assessment of communication efficiency in terms of number of messages transmitted in the network (this is analysed in the validation and performance evaluation section).

### Network exploration

In this stage of the *MOSAIC *process, the MCP asks the YP to obtain the list of MCC to whom the data access requests can be addressed. The reference of the MCC delivered by the YP are those hosting a dataset of the type requested by the MCP. For each MCC three situations may arise:

• **No constraint **The MCC offers the requested data set with no constraints to fulfil through a UCC. The UCP receives the notification of the data set availability and notifies this to the MCP.

• **Constraint resolved locally **The constraint requested by the MCC can be delivered from the dataset owned by the node (i.e. without the need of an external MCC). The UCP asks to its MCP to look for the MCC active in its node in order to collect the data from its DataMart. The UCP sends the notification of the dataset availability at the UCC after the potential fulfilment of the constraint. The UCC sends the agreement for the possible dataset transfer initially requested to the UCP. Both UCC and UCP notify their agreement for the potential exchange of the corresponding datasets to their MCC and MCP.

• **Constraint to be resolved externally **The constrain of the MCC cannot be resolved locally at the requesting node of the MCP. If the length of the path does not exceed the limit (monitored through a Time To Live -TTL- parameter) The UCP launches a new MCP to look for the data set needed in order to solve the constraint.

**Figure 3:**
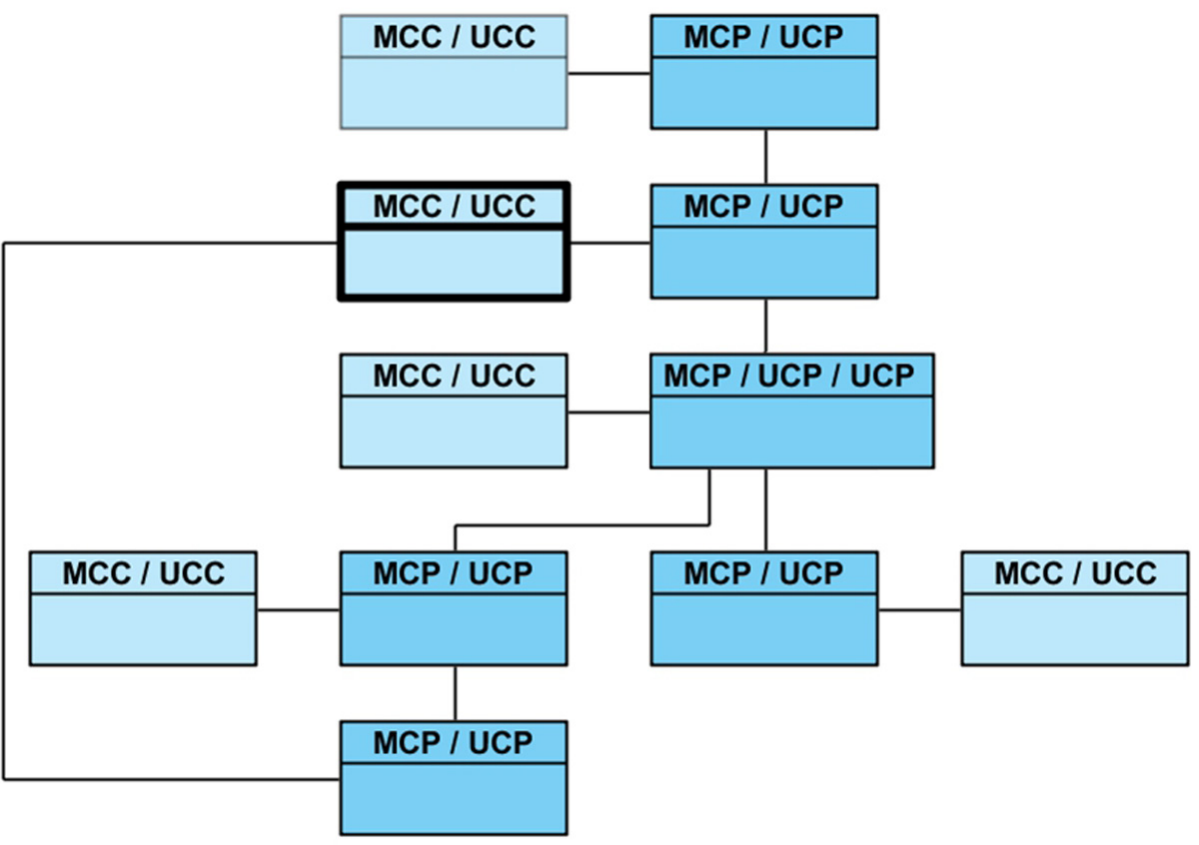
**Network path**. Example of a path in the network exploration where a MCC (in bold) will deliver its dataset without solving its constraint after identifying a loop.

A node and an MCC can take part more than once in a path of a multilateral agreement, however a special case occurs when in order to solve a constraint of an MCC the subsequent activations of new MCP results in a new request to the same MCC. If the request comes from an MCP "child" (belonging to the same branch), the MCC decides to activate the UCC without any constraint and thus, deliver its dataset without receiving any dataset in advance (see Figure 3). After completing the delivery of the other datasets in the path links the MCC receives the dataset of its constraint from the first MCP of the branch that initiated the negotiations.

The exploration of the network may cover all the possible paths (flooding) or a selection of them. The use of flooding is not only inefficient, but not feasible due to the computational costs. Therefore, when an MCP receives the set of MCC candidates, it will select a subset of them to continue the network exploration. The goals for an intelligent selection of the path are: i) get as much data as possible from the network, ii) get the most appreciated data (ranked with higher quality marks) and iii) reduce the risk of agreement failure (rollback).

For the first evaluation of the protocol, the criterion of the path selection chosen in this version of the protocol was the size of the dataset hosted at the MCC node. This criterion has been compared with the random selection of an MCC among the list of candidates and the results are described in the evaluation of the protocol (Validation and performance evaluation section). There is however, a set of indicators and more elaborate strategies that can be used for this purpose:

• **Agreement Reputation **Calculated at MCC level and based on the number of previous exchange agreements and those where the MCC has fully respected its commitments.

• **Dataset Reputation **Calculated also at MCC level, and based on the mean of the score given by each MCP, ranking the data delivered by a MCC after a successful data exchange agreement. Specific indicators that could be used are: i) Dataset size (number of items transmitted) and ii) Dataset cost (value paid for the items exchanged).

• **On-line Network Analysis **A dynamic classifier calculated also at MCC level, and based on stream data mining techniques, updated in real-time according to the dynamic behaviour of the negotiation process. This strategy will learn from the experience and the successful or failed attempts to reach a dataset after after every request.

The *MOSAIC Manager *permits to adjust the network exploration according to the user preferences and the specific density, inclusiveness, or degree of distribution of the network.

When an MCP does not find any MCC with the data set needed to fulfil a constraint, it stops the exploration and notifies to its creator UCP on the failure of the path in its attempt to find a multilateral agreement.

The network exploration concludes when all the paths: i) have concluded successfully and there is a possible multilateral agreement or ii) have failed in the agreement exploration or iii) the length has exceeded the TTL limit.

The complexity and performance of *MOSAIC *depends on the strategy and behaviour of the Agents, mainly set by two parameters: The number of branches allowed to explore by every *MCP *corresponding to the number of MCC activated (N) and the maximum lenght of the paths (TTL). The computational cost grows exponentially as bigger N and TTL (see Eq. (2)), and this could be compared with the complexity of the Dijkstra algorithm, which is *O(E + VlogV *), where *V *is the number of vertex and *E *the number of edges, but as indicated in the introduction, this approach for finding the shortest path can be only used when the topology of the network is known.

(2) $$NumMCP_{r}=\overset{TTL}{\underset{n=1}{\sum }}N^{TTL}$$

## The MOSAIC implementation

The interaction between the actors of *MOSAIC *respects the following principles: i) The users of the protocol interact with the Multicast Agents, ii) Unicast Agents are created by Multicast Agents to negotiate every possible data exchange between two nodes, and iii) Multicast Agents communicate with Multicast Agents, Unicast agents with Unicast Agents, Petitioner Agents with Petitioner Agents, and Contributor Agents with Contributor Agents. Direct communications between an MCP and a UCC or between an MCC and a UCP are avoided.

Two important aspects of the implementation correspond to i) the way that a path of a possible agreement is created and propagated and ii) the way a loop is detected.

The MCP has been implemented according to Algorithm 1, the MCC is presented in Algorithm 2, the UCC in Algorithm 3, and the UCP implementation is presented in Algorithm 4. In order to clarify the process and to highlight only the most important features of the protocol, the pseudocode presented here merges the steps of stages 2 to 5 of the negotiation process and after an agreement, the dataset is directly transfered to the requesting agent.

### Agreement paths

After the activation of a new request by the user a *Request *object is created. An instance of this object will be linked to every UCP and includes i) the ID of the requesting node, ii) the ID of the first MCP Agent of the negotiation chain, and iii) the ID of the negotiating branch. The value of the ID of the negotiating branch corresponds to a list of numbers that increases at every step of the path creation. When an MCP is launched by another MCP it receives from its creator the *Request *object and adds to the branch ID a new number. In doing so the *Request *object will contain the information needed to create the agreement paths.

A UCP arrives to the end of a path candidate to solve a multilateral agreement, when it receives the requested data from its UCC without the need to launch any other MCP. Consequently, it creates a message that will represent the negotiation path to which the UCP belongs to. This object is propagated to the higher levels of the Petitioners chain up to the MCP that initiated the request. During this bottom up process of transferring the agreement path candidate, all the Petitioners, at every link of the path, add to the object the relevant information and reference of the nodes to which there is a possible agreement. These correspond to the nodes where the MCC participating in the negotiation process with every MCP are hosted. At the end of the process of network exploration, the MCP that initiated the request receives, for every dataset of interest, the set of negotiation paths that correspond to a possible multilateral agreement. At that point, the MCP decides which negotiation paths to select from the possible candidates. An initial selection is performed among the paths that arrive to the same dataset, but the MCP may also decide to execute only a subset of all the remaining negotiation path candidates, based on other criteria (e.g. cost or reputation).

**Algorithm 1 **Multicast Petitioner Agent (*MCP *)

Inputs

*ResourceRequested *from *User *or *UCP*

*NegotiationAgreement *from *UCP*

*ResourceDataset *from *UCP*

*MCC *from *YellowPages *(YP)

1: Ask *Y P *for MCC hosting the *ResourceRequested*

2: Collect *MCC *compatible from *YellowPages*

3: Select *MCC *to negotiate

4: **for all ***MCC *selected **do**

5:     Create *UCP (ResourceRequested*)

6:     Ask the *UCP *to start the negotiation

7:     **if ***NegotiationAgreement = TRU E ***then**

8:        Collect *ResourceDataset *from *UCP*

9:        Send *ResourceDataset *to the *User *or *UCP*

10:     **end if**

11: **end for**

**Algorithm 2 **Multicast Contributor Agent (*MCC*)

Inputs

*ResourceOf fered *from the *User*

*Constraint *from the *User*

*Request *from the *MCP*

*NegotiationAgreement *from the *UCC*

*ConstraintDataset *from the *UCC*

1: Add *MCC *to the *YellowPages *(YP)

2: **while **User does not stop the *MCC ***do**

3:     Get *Request *from some *MCP*

4:     **if ***Request = ResourceOffered ***then**

5:         **if **Child-Loop detected **then**

6:             Create *UCC(Request, NUL*)

7:         **else**

8:             Create *UCC(Request, Constraint*)

9:         **end if**

10:         Ask the *UCC *to start the negotiation

11:         **if ***NegotiationAgreement = TRUE ***then**

12:             Collect *ConstraintDataset *from *UCC*

13:         **end if**

14:     **end if**

15:     Remove *UCC*

16: **end while**

17: Remove *MCC *from the *YP*

**Algorithm 3 **Unicast Contributor Agent (*UCC*)

Inputs

*Request *from *UCP*

*Constraint *from *MCC*

*ResourceOf fered *from *MCC*

*ConstraintDataset *from *UCP*

*ConstraintSolved *from *UCP*

1: **if ***Constraint ≠ NUL ***then**

2:     Send *ResourceOffered *to UCP

3:     *NegotiationAgreement ← TRUE*

4: **else**

5:     Ask the *UCP *to solve the constraint

6:     **if ***ConstraintSolved = TRUE ***then**

7:         Collect *ConstraintDataset *from *UCP*

8:         Send *ConstraintDataset *to *MCC*

9:         *NegotiationAgreement ← TRUE*

10:     **else**

11:         *NegotiationAgreement ← FALSE*

12:     **end if**

13: **end if**

14: **return ***NegotiationAgreement*

**Algorithm 4 **Unicast Petitioner Agent (*UCP *)

Inputs

*ResourceRequested *from *MCP*

*constraint *from *UCC*

*constraintDataset *from *MCC*

1: Ask the *UCC *to send the *ResourceRequested*

2: **if ***Constraint *≠ *NUL ***then**

3:     Search *MCC *in the Node to solve the constraint

4:     **if ***MCC *≠ *N U L ***then**

5:             *ConstraintSolved ← TRUE*

6:             Get *ConstraintDataset *from *MCC*

7:             Send *ConstraintDataset *to the *UCC*

8:     **else**

9:         Create *MCP *to look for the *ConstraintDataset*

10:         **if ***ConstraintDataset *found **then**

11:             *ConstraintSolved ← TRUE*

12:             Send *ConstraintDataset *to the *UCC*

13:         **else**

14:             *ConstraintSolved ← FALSE*

15:             Notify failure to solve the constraint to the *UCC*

16:         **end if**

17:     **end if**

18: **end if**

19: **if ***Constraint = N U L ***or ***ConstraintSolved ***then**

20:     Collect *ResourceRequested *from the *UCC*

21:     Send *ResourceRequested *to *MCP*

22:     *NegotationAgreement ← TRUE*

23: **else**

24:     *NegotationAgreement ← FALSE*

25: **end if**

26: **return ***NegotationAgreement*

### Loop detection

Each agreement path or branch of the *Petitions Tree *is built during the network exploration. Every branch is identified with a *request identifier *corresponding to an array where each of its elements represents the participation of a Petitioner in the branch. It is important to note that an MCP will belong to more than one branch when i) it has more than one UCP exploring different options of agreement or ii) there is another MCP in the lower levels of its path with the same situation (managing more than one UCP).

A new request received by an MCC is processed and compared with all the other active requests managed by the MCC.

A loop is identified when all the elements of the array of some request identifier, that are active in the MCC, is equal to the first elements of the request identifier of the new request received, which means that the request comes from the same branch of that already active request at the MCC. In that case, the associated UCC will be created without any constraint. Security issues that arise here have already been studied and analysed [20].

## Validation and performance evaluations

The assessment performed to the *MOSAIC *system includes the validation and evaluation of i) the correctness of the protocol, ii) the advantatges of multilateral agreements compared with bilateral ones; iii) the optimisation process for the network exploration; abd iv) the analysis of the type of nodes that most benefit from the *MOSAIC *system.

### The scenario evaluation

COPD comorbidities include a large list of diseases (see Table 1). For a better prognosis of a certain patient, analysing the information collected from other patients with similar profiles and suffering the same comorbidities, may be relevant.

**Table 1:** Datasets of the scenario evaluation: The most common comorbidities of COPD

Dataset	COPD Comorbidity	Number of cases
D01	Peripheral arterial diseases	3663
D02	Prostate cancer	1242
D03	Chronic renal failure	4747
D04	Osteoporosis	1097
D05	Gastroesophageal reflux disease	5194
D06	Hyperlipidemia	37890
D07	Congestive heart failure *	3184
D08	Obstructive sleep apnea	1386
D09	Atrial Fibrilation/Flutter *	3011
D10	Lung cancer	1135
D11	Erectile dysfunction	2251
D12	Depression	4963
D13	Breast cancer	511
D14	Pulmonary fibrosis *	236
D15	Substance abuse	3169
D16	Abdominal aortic aneurism *	404
D17	Anxiety *	2257
D18	Benign prostatic hypertrophy	19600
D19	Hypertension	77908
D20	Hypothyroidism	667
D21	Gastric/duodenal Ulcer *	1882
D22	Diabetes	5380
D23	Psychiatric disorders	1092
D24	Coronary artery disease *	29806
D25	Pulmonary HTN+CP *	1599
D26	Cataract	194
D27	Cerebrovascular accident	1814
D28	Degenerative joint disease	10373
D29	All others	131
	**TOTAL**	**226791**

Moreover, the knowledge of the effect of certain therapies to other patients with similar profiles may help the clinician to provide a more effective and efficient treatment to his or her patients. The *MOSAIC *system could help both prognosis and theragnosis by facilitating the multilateral agreements for data exchange.

In this framework, the scenario used to test the *MOSAIC *system, is composed by a set of nodes each of them with a number of datasets corresponding to a comorbidity of COPD. For each dataset each node activates an MCC. Every MCC is associated to a constraint corresponding to a disease randomly selected from all possible comorbidities or - with the same probability as any data type - to an empty constraint, in which case the MCC freely offers its dataset to any MCP. Two datasets have been created: One with 2.852 nodes corresponding to the main cities around the world, hosting 18.902 data sets; and another one for the most complex and time consuming evaluations with a subset of 205 cities with 1.824 datasets.

The evaluation of *MOSAIC *has been performed on this simulated, but realistic scenario. The results shown in this article are based, firstly on the activation of the MCC for the datasets with the same cases available, and secondly on the activation of a request (or MCP) for every possible dataset, by every node (or city). This corresponds to 2.852 × 28 = 79.856 requests for the whole worldwide network and to 205 × 28 = 5.740 requests for the smaller network.

### The database of the evaluation scenario

The evaluation scenario corresponds to a set of nodes (cities) each hosting a number of datasets with clinical cases of people suffering COPD and some comorbidities. While some datasets are freely offered to the network without any restriction, most of them have a constraint associated, requiring the delivery of some other dataset that may be available at some other node. The data base created for the simulations (freely available upon request) is composed by a network of 2.852 nodes corresponding to cites worldwide distributed with 18.902 datasets in total.

The constraints have been simulated calculating a random figure (from 0 to 29) at every node for every dataset. '0' represents that there is no dataset available with a COPD patient suffering from a specific comorbidity and no constraint can be assigned for delivering nothing. Any number between '1' and '28' indicates the reference of the COPD comorbidity to be delivered by the requesting node (as constraint for authorising the access to the data). '29' indicates that there is no constraint to fulfill and the cases available at the node for that specific comorbidity will be freely delivered to the requesting node.

On one hand, nodes with a large number of cases covering most of the data types will have a higher chance to directly solve a possible constraint and achieve bilateral agreements. On the other hand, the nodes with a reduced number of cases in their datasets will likely need multilateral agreements to get the data desired from the network. One of our hypothesis is that the system presented here will be especially useful for nodes with less chances to achieve bilateral agreements.

### Simulation output

After the simulation execution a DB with the total number of cases collected per node and datatype was created. Table 2 shows a subset of that database after the execution of the simulator with TTL = 20. Its content corresponds to the following:

• **Node**: Requesting node

• **R**: Resource requested

• **C**: Initial number of cases of type R at the requesting node

• **MCP**: Number of MCP participating in the multilateral agreement

• **MSG**: Number of messages exchanged

• **CC**: Number of new cases of type R collected from the network

• **Path**: Average length of the multilateral agreement path

All the results presented in this article have been obtained after the processing of these figures.

**Table 2:** Data Base generated after the protocol execution

Node	R	C	MCP	MSG	CC	Path
Akron	D01	0	2	20	10	3
Akron	D02	0	6	156	10	7
Akron	D03	10	2	20	70	3
Akron	D04	0	8	272	10	9
...						

### Evaluation results

A first evaluation of the *MOSAIC *protocol is a cross validation to check that it works properly. For this, algorithm 5 has been created. It scans the network of 2.852 cities and their 18.902 data sets seeking for all possible bilateral agreements. This algorithm generates as an output a matrix with the figures corresponding to all the cases collected by each node for every data type after the bilateral data exchanges. These figures have been compared with those obtained by the execution of the *MOSAIC *protocol with the Time to Live parameter set to 1, forcing that the maximum length of every multilateral agreement is limited to 2 nodes. The results obtained in both cases are exactly the same, showing the correctness of the protocol for this scenario.

**Figure 4:**
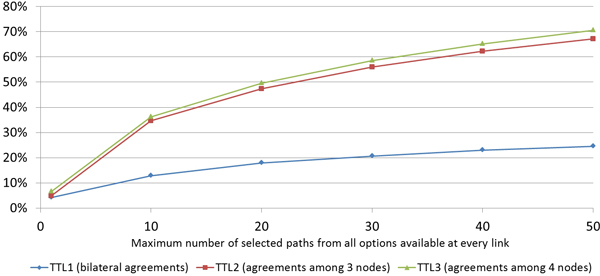
**Cases collected from the network**. Percentage of cases collected from the total number available in the network with different values of TTL and size of the selected path set.

The second evaluation is to prove the main goal of the *MOSAIC *system which is to overcome the amount of data that can be exchanged with bilateral agreements and collect as much data as possible from the network by achieving as much data exchange agreements as possible. The results obtained strongly depend on the parameters of the protocol, namely its TTL and the number of branches selected from all the paths available during the network exploration. Figure 4 shows the results with different values for TTL (1: bilateral agreements, 2: agreements among 3 nodes, and 3: agreements among 4 nodes) and with a range selection of paths starting from 1 (only exploring a single MCC from all available) to 50 (higher values of TTL are not needed as most of the data available in the network is made accessible after much more short negotiation paths). The percentages of data collected shows a steady increase when the selection of the number of possible paths increases, and while the improvement from TTL1 to TTL2 is significant, the increase from TTL2 to TTL3 is limited.

**Algorithm 5 **Search for bilateral exchange agreements

1:   **for ***i *= 1 to *numCities ***do**

2:     **for ***j *= 1 to *numDataTypes ***do**

3:       **for ***k *= 1 to *numCities ***do**

4:         **if ***i/ = k ***then**

5:           **if ***Dataset[k, j*] ≠ 0 **then**

6:             **if ***constraint[k, j*] = *nul ***then**

7:               collect *Dataset[k, j*] for node *i*

8:             **else if ***constraint[k, j*] available in node *i ***then**

9:               solve *constraint *from data in node *i*

10:               collect *Dataset[k, j*] for node *i*

11:             **end if**

12:           **end if**

13:         **end if**

14:       **end for**

15:     **end for**

16: **end for**

Due to the time constraints during the simulations of the protocol behaviour with different TTL values, a subset of the whole DB has been created. Only 205 cities of the initial DB have been used for the simulations.

The third evaluation refers to the optimisation process for the network exploration through the intelligent selection of the paths to follow. As indicated in the *MOSAIC *implementation section, the MCP receives the list of MCC compatible from the Yellow Pages and in order to avoid unmanageable network explorations the MCP has to decide which to select and which to discard. Two cases have been evaluated. One selects a MCC randomly from those available, and the other selects the MCC with the biggest dataset. The two cases have been tested using the database of 205 cities with 1.824 datasets in total.

**Figure 5:**
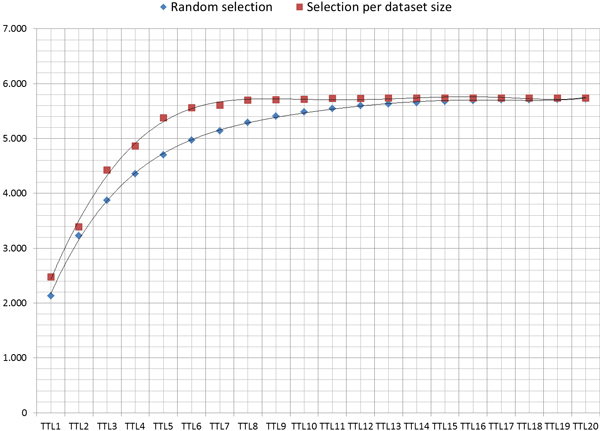
**Agreements**. Total number of agreements using *MOSAIC *with different TTL values.

Figure 5 shows the improvement in the number of agreements, when selecting the path to follow during the network exploration according to the size of the MCC dataset, instead of a random selection among the MCC available. When an MCP child (belonging to the same branch) needs to resolve a set of constraints to obtain the desired dataset, it is more likely to have an MCC in the higher levels of its branch that can solve it when the MCC's dataset is bigger. In those cases, the MCC that will receive a data access request from a child node (belonging to the same branch) will decide to offer the requested dataset to solve the constraint as this loop will benefit the overall multilateral agreement and the MCC will also get the desired dataset initially included as a constraint.

**Figure 6:**
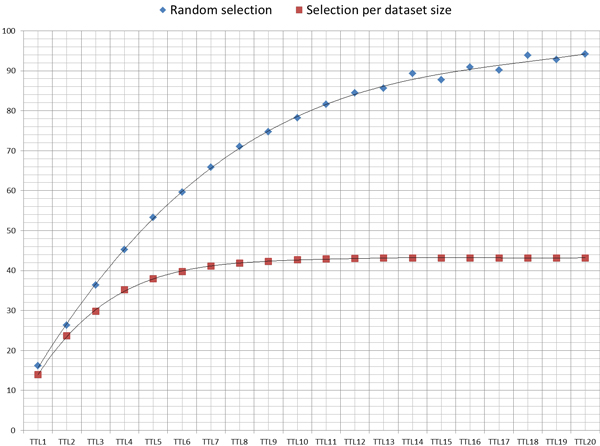
**Messages**. Comparative of the average number of messages needed to achieve an agreement, between the two branch selection strategies.

Figure 6 shows the reduction in the number of messages transmitted over the network needed to achieve an agreement, comparing the selection criteria of the MCC between the strategy based on the dataset size and the random selection.

**Figure 7:**
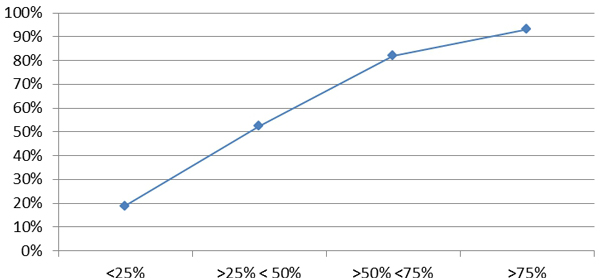
**Cases collected through bilateral agreements**. Average of the percentage of cases collected by the nodes after bilateral exchanges. Nodes have been grouped according to the relative size of their local Data Marts in four categories. As larger Data Marts, as more data collected.

Finally, we checked our hypothesis that the nodes with less data which had less chances to achieve bilateral agreements would be those that specially benefit from *MOSAIC*. The figures corresponding to this hypothesis are shown in Figure 7 and are generated after running the *MOSAIC *protocol in the evaluation scenario of the worldwide network. The total set of nodes has been grouped in 4 categories according to the size of the datasets (e.g. "less than 25%" indicates the category of the set of nodes with a number of cases in their datasets, minor than the 25% of the average size of all datasets in the network).

## Conclusions and future work

It has been demonstrated that the multilateral agreements among a set of nodes increase significantly the amount of data accessible in a network compared with the amount of data that can be collected from bilateral agreements.

Besides this, the need of a distributed process to support the achievement of multilateral agreements has been justified for the lack of global knowledge of the network topology derived from the reluctance to publish certain information in a centralised repository. The use of Agents has facilitated to model the negotiation process required by the actors of this system and this seems a natural way to implement the protocol.

It has been proved that the strategy to select the path to follow during the exploration of the network has implications in the number of agreements achieved among the nodes. For this, two criteria have been tested: i) A random selection and ii) A selection based on the Dataset size. Finally, it has been demonstrated that the total number of agreements among the nodes achieve better marks when the path selection is based on the dataset size.

### Future work

The research presented here is being extended or it is planned to be extended in the following aspects:

• **Semantic representation**. Both datasets and constraints represented using OWL, as the standard for knowledge semantic representation.

• **Constraints enrichment**. A more natural representation of the possible constraints will be based on a boolean expression composed by a set of clauses, some of them related to the delivery of a combination of certain datasets (not only a single one) and others related to the acceptance or rejection of certain top level conditions for the data access by the user.

• **Core implementation**. The optimisation of the code to allow wider and deeper path explorations of the network in a reasonable time and the visualisation of the protocol results in a web based interface.

• **Security and privacy**. Data disclosure protection, attacks prevention, authenticity, and other features of privacy and security are issues that will be integrated in the protocol with the deployment of previous research in the field [21,22] and their adaptation to this specific scenario.

• **System deployment and evaluation in different scenarios**. It is expected that the results of the protocol will differ significantly depending on the specific scenario and characteristics of the network. Therefore, it is also planned to adapt the behaviour of the Agents to different frameworks and to identify which strategies are the best for each case and specifically the best balance between path length and branch selection wide (number of branches to explore among all the possible).

• **Intelligent exploration**. Increase the intelligence of the path selection by including more advanced indicators (e.g. reputation, user similarity and cost) considering also previous research in the areas of agent trust, argumentation and reasoning [23,24].

• **Game theory**. During the network exploration the MCP launches a request to get the desired data or to solve a constraint (if it is not the first MCP in the path). A set of MCC may answer and one or a set of them have to be selected.

On the one hand, the selection of the best MCC depends on the decision of the set of nodes or MCC-MCP pairs already involved in the agreement. All of them share the goal to complete the path and achieve the multilateral agreement with the new MCC that has to be selected. All of them want to maximise the chances to achieve a successful agreement and will share the information to get the best decision. On the other hand, the set of MCC candidates compete among them to be selected and be part of the multilateral agreement.

This can be modelled as an auction where the buyers are the set of participants at the partial path already built and the sellers are the MCC candidates. The MCC compete among them, but they could also agree among them some strategy and build some coalition in order to overcome their rivals.

## Competing interests

The authors declare that they have no competing interests.

## Authors' contributions

M. Lluch-Ariet conceived and designed the MOSAIC System. J. Pegueroles-Vallés coordinated the scientific advances of the research and provided the top level guidelines to boost the results, together with F. Vallverdú. The implementation of the system has been done together between A. Brugués de la Torre and M. Lluch-Ariet.

## Declarations

This research (including this publication) has been partially funded by the Synergy-COPD research grant, under the Seventh Framework Program of the European Commission as a Collaborative Project with contract number 270086 (2011-2014), and by the project TAMESIS (TEC2011-22746).

This article has been published as part of *Journal of Translational Medicine *Volume 12 Supplement 2, 2014: Systems medicine in chronic diseases: COPD as a use case. The full contents of the supplement are available online at http://www.translational-medicine.com/supplements/12/S2.
